# Methodology Validation: Correlating Adherent Scalp Flaking Score (ASFS) With Phototrichogram for Scalp Dandruff Evaluation in Adult Subjects

**DOI:** 10.7759/cureus.63247

**Published:** 2024-06-26

**Authors:** Maheshvari N Patel, Nayan K Patel, Apeksha M Merja, Dhruvil U Gajera, Arnav K Purani, Jemini H Pandya

**Affiliations:** 1 Clinical Research, NovoBliss Research, Ahmedabad, IND; 2 Pharmacology, Swaminarayan University, Ahmedabad, IND; 3 General Medicine, NovoBliss Research, Ahmedabad, IND; 4 Dermatology, NovoBliss Research, Ahmedabad, IND

**Keywords:** multirater kappa test, inter-evaluator variability, phototrichogram, adherent scalp flaking score, pityriasis capitis

## Abstract

Introduction

Scalp dandruff is a common dermatological condition characterized by flaking and itching of the scalp, affecting a significant portion of the population. Effective assessment methods are crucial for evaluating treatment outcomes. This study aimed to establish the reliability and correlation of three assessment techniques: Adherent Scalp Flaking Score (ASFS), phototrichogram using CASLite Nova, and the 60-second hair combing test.

Methods

This open-label, single-arm, single-center, prospective clinical study enrolled 12 adult subjects with mild to moderate dandruff. Evaluations were conducted before and after a standardized hair-wash intervention using three methods: ASFS, phototrichogram using CASLite Nova, and the 60-second hair combing test. The primary objective was to establish correlations between these assessment techniques. Inter-evaluator and inter-operator reliability were assessed using Fleiss Multirater Kappa.

Results

Significant reductions in dandruff were observed across all methods post-hair wash. The ASFS decreased from 23.67±2.06 at baseline to 6.67±4.46, showing a mean reduction of 17.00±5.22 (71.40%, p<0.001). phototrichogram analysis revealed that 60.42% of the total (n=96) scalp zones assessed were in normal condition post-hair wash compared to none at baseline. The 60-second hair combing test showed a reduction in non-adherent flakes, with 58.33% of subjects displaying light flakes and 41.67% showing no flakes post-hair wash. The chi-squared test indicated a significant association (p<0.001) between ASFS and phototrichogram results. Inter-evaluator variability for ASFS and the hair combing test demonstrated substantial agreement (Kappa=0.692 and 0.637, respectively, p<0.0001). Inter-operator reliability for phototrichogram also showed substantial agreement (Kappa=0.746, p<0.0001).

Conclusion

The study confirms the reliability and consistency of ASFS, phototrichogram, and the 60-second hair combing test in assessing scalp dandruff. The significant correlations of ASFS and phototrichogram via CASLite Nova validate their use in clinical settings. Comprehensive training for evaluators and operators is essential to achieve reproducible and accurate results. These findings provide a robust framework for future studies and clinical assessments of scalp dandruff.

## Introduction

Scalp dandruff, also known as pityriasis capitis or seborrheic dermatitis, is a common dermatological condition characterized by the shedding of skin cells from the scalp. It presents as white or yellowish flakes on the scalp and often leads to itching and discomfort. This condition affects a significant portion of the population worldwide, with estimates suggesting that up to 50% of adults experience dandruff at some point in their lives. Despite its prevalence, the exact etiology of scalp dandruff remains unclear, with factors such as excessive sebum production, fungal colonization by Malassezia species, and individual susceptibility playing potential roles [1-5].

Dandruff can manifest in two primary forms: adherent and non-adherent. Adherent dandruff refers to flakes that tightly adhere to the scalp, often requiring physical manipulation to remove, while non-adherent dandruff consists of loose flakes that easily detach from the scalp and are visible on the hair or clothing. Understanding the distinction between these two types is crucial for developing effective treatment strategies and assessing treatment outcomes. Various methods exist for assessing scalp dandruff, each with its strengths and limitations. Traditional clinical assessments, such as visual inspection and dermatological scoring systems like the Adherent Scalp Flaking Score (ASFS), provide subjective evaluations of dandruff severity based on the presence and extent of adherent flakes. On the other hand, instrumental techniques, such as scalp phototrichogram using devices like CASLite Nova, offer objective measurements of scalp condition by analyzing parameters such as hair density and follicle health. Additionally, non-invasive tests like the 60-second hair combing test provide a quick and practical way to assess the amount of non-adherent flakes present on the scalp [6-10].

In this study, we aimed to address the need for comprehensive evaluation and correlation of different assessment methods for scalp dandruff. Specifically, we utilized three distinct techniques: Adherent Scalp Flaking Scoring by dermatologists, scalp phototrichogram using CASLite Nova, and the 60-second hair combing test. Each method offers unique insights into dandruff severity and scalp condition, contributing to a holistic understanding of the condition. Given the variability observed in evaluations conducted by different raters and operators, establishing standardized methods is essential to ensure the reliability and consistency of dandruff assessments. To address this challenge, we performed a Fleiss Multirater Kappa test to quantify inter-evaluator and inter-operator variability and post-comprehensive training by dermatologists and instrument experts, thereby, providing a standardized framework for future assessments [11].

The primary objective of this study was to establish correlations between assessments for dandruff made using the ASFS, scalp phototrichogram, and 60-second hair combing test before and after hair wash interventions in adult subjects with scalp dandruff. By elucidating the associations between these assessment methods, we aimed to provide valuable insights into the reliability and validity of each technique for evaluating scalp dandruff severity. Additionally, we sought to evaluate the variability observed in assessments conducted by different evaluators and operators, emphasizing the importance of standardizing evaluation methods to enhance the reproducibility of results. Through rigorous statistical analysis and methodological validation, we aimed to contribute to the scientific understanding of scalp dandruff assessment and pave the way for improved diagnostic and therapeutic approaches in clinical practice.

## Materials and methods

Ethical conduct of the study

This study was conducted in accordance with the principles outlined in the Declaration of Helsinki and the ICH Good Clinical Practice and the Indian Council of Medical Research guidelines. Ethical approval for the study plan was obtained from the Ethics Committee prior to the commencement of any study-related activities. All participants provided signed informed consent before enrolment in the study. The consent process included a detailed explanation of the study objectives, procedures, confidentiality measures, and the voluntary nature of participation.

Furthermore, this clinical study was registered with the Clinical Trial Registry of India (CTRI) [CTRI/2024/05/067755] and ClinicalTrials.gov. This comprehensive ethical framework ensures the study's compliance with international and national ethical standards, safeguarding the rights, safety, and well-being of all participating subjects.

Study design

This open-label, single-arm, single-center, prospective clinical study aimed to validate the methodology of scalp dandruff assessment using multiple techniques. The study was conducted at NovoBliss Research Private Limited, Ahmedabad, India, involving a total of 12 adult subjects aged 18 to 55 years with mild to moderate scalp dandruff. The primary objective was to establish correlations between assessments for dandruff using the ASFS, scalp phototrichogram using CASLite Nova, and the 60-second hair combing test, both before and after hair wash interventions. The secondary objective was to establish inter-operator and inter-evaluator reliability for these assessment techniques. Subjects were screened based on predefined inclusion and exclusion criteria to ensure a homogeneous study population. Inclusion criteria included healthy adult males and non-pregnant, non-lactating females in good general health, with no history of adverse skin conditions or medications likely to interfere with the study results. Exclusion criteria ruled out unwilling participants and those with allergies to dermatological or cosmetic products.

**Figure 1:**
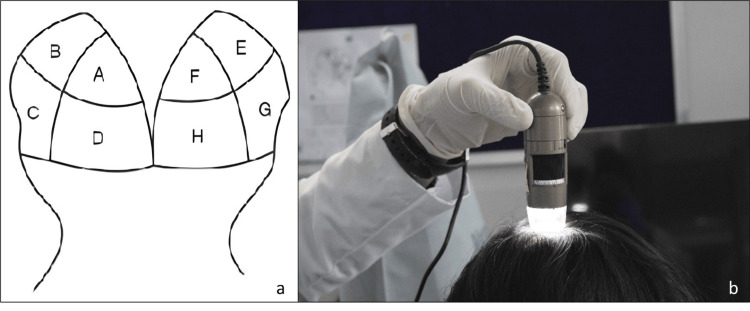
(a) Division of scalp into eight sections for ASFS grading; (b) capturing phototrichogram using CASLite Nova

The study procedures were conducted on a single visit (Day 1), divided into pre- and post-hair wash assessments. The pre-hair wash assessments included screening and enrolment, followed by evaluations using ASFS, scalp phototrichogram via CASLite Nova, and the 60-second hair combing test. Each subject's scalp was divided into eight zones for ASFS evaluation as shown in Figure 1a, where each zone was scored on a scale from 0 to 10 based on the presence of adherent flakes. The total ASFS score, ranging from 0 to 80, was then categorized into mild, moderate, and severe dandruff as follows: 1=mild (16-24), 2=moderate (25-34), and 3=severe (35-80).

**Figure 2:**
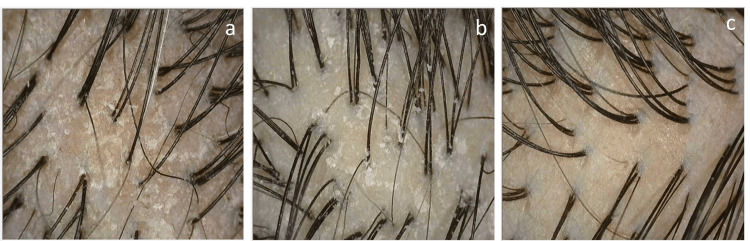
(a) Dry scalp with much keratin; (b) dry scalp with some keratin; (c) normal scalp with good condition hair thickness and density

Scalp phototrichograms were captured using CASLite Nova at the same sites marked for ASFS zones. Images were taken at a magnification of 60x, ensuring clear visualization of the scalp. Consistent lighting conditions were maintained across all evaluations. The captured images were analyzed by comparing them with reference images from the CASLite Nova software, which depict various scalp conditions. Figure 2 shows the reference images for different scalp conditions. This technique was employed to assess the scalp condition of all eight zones accurately, as shown in Figure 1b. The 60-second hair combing test involved instructing subjects to comb their hair over a black cloth placed on their shoulders for 60 seconds. A fine-toothed comb was used to gently comb the hair in the direction of its growth. The black cloth used was a regular barber cape covering the subject’s shoulders and back. A dermatological evaluation was then conducted to assess visible flakes fallen, scored on a four-point scale from 0 (none) to three (heavy flakes).

After completing the pre-hair wash assessments, subjects underwent a hair wash using a standard marketed shampoo. The shampoo used for hair wash was "Dove Anti-Dandruff Shampoo." The hair wash procedure was consistent for all participants. The shampoo application was done for five minutes, twice, consecutively, with a focus on the scalp, followed by rinsing under running water. Post-hair wash evaluations mirrored the pre-hair wash procedures and were performed by the same evaluator to reduce variability. Furthermore, to reduce bias, the evaluators were blinded to the subjects' pre-wash conditions during the post-wash assessments. This blinding was achieved by ensuring that the evaluators did not have access to the pre-wash assessment data or any identifying information about the subjects during the post-wash evaluations. Photographic documentation was performed to capture visual evidence of scalp condition and dandruff before and after the hair wash.

To address the secondary objective, inter-operator reliability was assessed by having five trained instrument operators take readings from the 12 subjects using CASLite Nova. These readings were compared to evaluate consistency across different operators. The training program for the instrument operators was conducted by a professional instrument expert. It included theoretical sessions on the principles and functions of CASLite Nova, practical hands-on training to familiarize operators with the equipment, and standardized procedures to ensure consistent data collection. The training emphasized the importance of maintaining consistent conditions such as lighting during image capture.

Inter-evaluator variability was measured by having one dermatologist, one physician, and four dermatologist-trained evaluators assess the same 12 subjects using ASFS and the 60-second hair combing test. The evaluators underwent a comprehensive training program led by the dermatologist. This program covered detailed theoretical knowledge about scalp dandruff and its clinical manifestations, practical sessions to standardize the assessment techniques, and calibration exercises to ensure uniform scoring criteria among evaluators. Fleiss Multirater Kappa was used to quantify the agreement among different raters, ensuring that the assessment techniques were reliable and reproducible.

Statistical analysis

The statistical analysis for this study was conducted using the Statistical Package for the Social Sciences (SPSS), Version 29.0.1.0(171). The analysis aimed to evaluate the primary and secondary objectives of the study, which included establishing correlations between different dandruff assessment methods and assessing inter-operator and inter-evaluator reliability. Continuous variables such as age and ASFS scores were described using descriptive statistics, including the number of observations (N), mean, standard deviation (SD), median, minimum, and maximum values. Categorical variables, such as the severity categories of ASFS scores (mild, moderate, and severe), were expressed as frequencies and percentages. Graphical presentations, such as bar charts and Sankey diagrams, were utilized to visually represent these categorical data distributions.

The Pearson chi-squared test was employed to examine the relationship between categorical variables. Specifically, it was used to determine whether there was a statistically significant association between the ASFS categories and the results from the 60-second hair combing test. The null hypothesis for the Pearson chi-squared test posited that there is no association between the variables, while the alternative hypothesis suggested a significant association. The chi-squared statistic was calculated using the formula: $x^2 = \sum \frac{(O_i - E_i)^2}{E_i}$; where Oi represents the observed frequency, and Ei represents the expected frequency under the null hypothesis. A p-value of less than 0.05 was considered statistically significant, indicating that the observed distribution of dandruff severity categories differed from what would be expected by chance.

The Fleiss Multirater Kappa was utilized to assess the reliability and consistency of the evaluations conducted by different raters. This statistical measure quantifies the level of agreement among multiple raters when classifying items into categories. The formula for Fleiss Kappa is $\kappa = \frac{\bar{P} - \bar{P}_e}{1 - \bar{P}_e}$; where $\bar{P}$ is the mean observed agreement among raters, and $\bar{P}_e$ is the mean expected agreement by chance. Fleiss Kappa values range from -1 to 1, where a value of 1 indicates perfect agreement; a value of 0 indicates no agreement beyond chance; a value less than 0 indicates less agreement than expected by chance (Table 1).

**Table 1:** Interpretation of Fleiss Kappa values κ: Kappa

Value of κ	0.01-0.20	0.21-0.40	0.41-0.60	0.61-0.80	0.81-1.00
Strength of agreement	Slight agreement	Fair agreement	Moderate agreement	Substantial agreement	Almost perfect agreement

Fleiss Kappa was used to assess both inter-operator reliability (comparisons among readings taken by different instrument operators) and inter-evaluator variability (comparisons among ratings given by the dermatologist, physician, and dermatologist-trained evaluators). High Fleiss Kappa values would indicate that the assessment methods are reliable and reproducible across different raters.

Data handling and analysis

All data were carefully reviewed and cleaned before analysis to ensure accuracy and completeness. Frequency analyses and cross-tabulations were performed to ensure data accuracy and consistency. Missing data were addressed through appropriate imputation methods. The results of the statistical tests, including p-values and Kappa coefficients, were reported with corresponding confidence intervals to provide a measure of precision and reliability.

Sample size determination

As this study was a pilot, proof-of-concept, methodology standardization, and inter-evaluator variability assessment study, and not a safety/efficacy assessment study or a randomized controlled trial, we employed convenience sampling, a non-probability sampling technique that involves selecting subjects based on their convenience and accessibility, in determining the sample size for our study. In our study, we confined our sample to the accessible population, which consisted of subjects with scalp dandruff visiting our study facility, who met the eligibility criteria. This approach allowed us to conduct a comprehensive analysis utilizing a small sample size to explore the feasibility and generate hypotheses for larger, more definitive studies.

Subject disposition

A total of 12 adult subjects aged 18 to 55 years were enrolled in this study. From the total of 20 subjects screened, six were excluded due to low levels of scalp dandruff, and two were excluded because of a history of allergic reactions to skin products. There were no dropouts or withdrawals, ensuring complete data collection for both pre- and post-hair wash assessments. The study maintained high compliance with the intervention and assessment schedules. The inclusion of both male and female subjects provided a representation, with 58.33% female and 41.67% male participants.

## Results

In our investigation of scalp dandruff assessment on 12 subjects, we used three evaluation methods: the ASFS, phototrichogram with CASLite Nova, and the 60-second hair combing test. Before and after a standardized hair-wash intervention, we observed changes in dandruff severity across all assessment techniques.

Changes observed post-baseline in assessment methods

**Figure 3:**
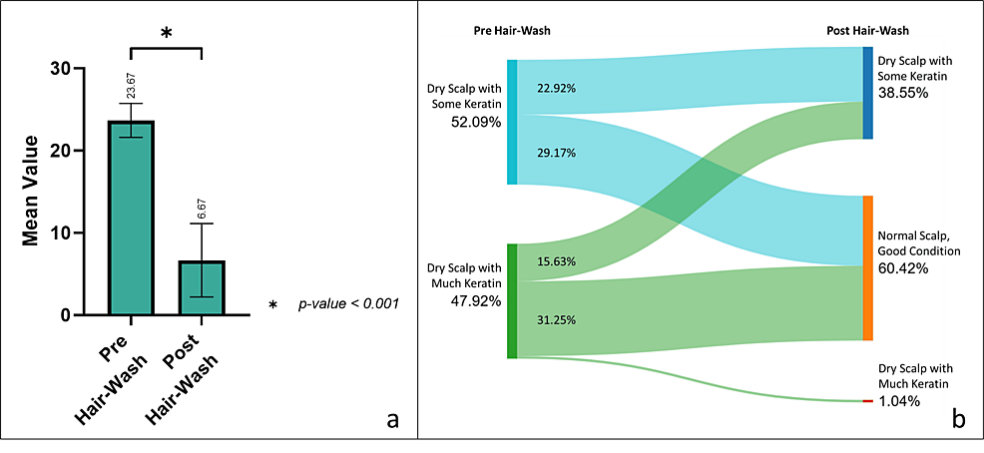
Change in adherent scalp dandruff; (a) ASFS scale assessed by dermatological assessment; (b) scalp condition assessed by phototrichogram using CASLite Nova

The intervention yielded notable changes across multiple assessment methods, highlighting the reliability and consistency of these techniques in evaluating scalp dandruff. The ASFS exhibited a substantial reduction from baseline to post-hair wash assessments, indicating a consistent decrease in adherent scalp flakes. At baseline, the ASFS was 23.67±2.06, significantly decreasing to 6.67±4.46 post-hair wash and drying (mean reduction: 17.00±5.22, 71.40% reduction, p<0.001). This reduction underscores the reliability of ASFS in quantifying adherent scalp flakes, a key characteristic of scalp dandruff (Figure 3a). Concurrently, scalp condition assessed using a phototrichogram by CASLite Nova demonstrated notable improvements post-intervention, with a significant proportion of scalp zones transitioning to normal, good condition. More than half of the scalp zones with some keratin and nearly two-thirds of the scalp zones with much keratin transitioned to normal, good scalp condition. Furthermore, post-intervention, 60.42% of total scalp zones assessed exhibited a normal and good condition, compared to none at baseline. The observed changes in ASFS values corresponded with the improvements in scalp condition post-intervention, suggesting a consistent trend in dandruff reduction across assessment methods (Figure 3b). The reference images of phototrichograms by CASLite Nova depicting the different scalp conditions are shown in Figure 2.

**Figure 4:**
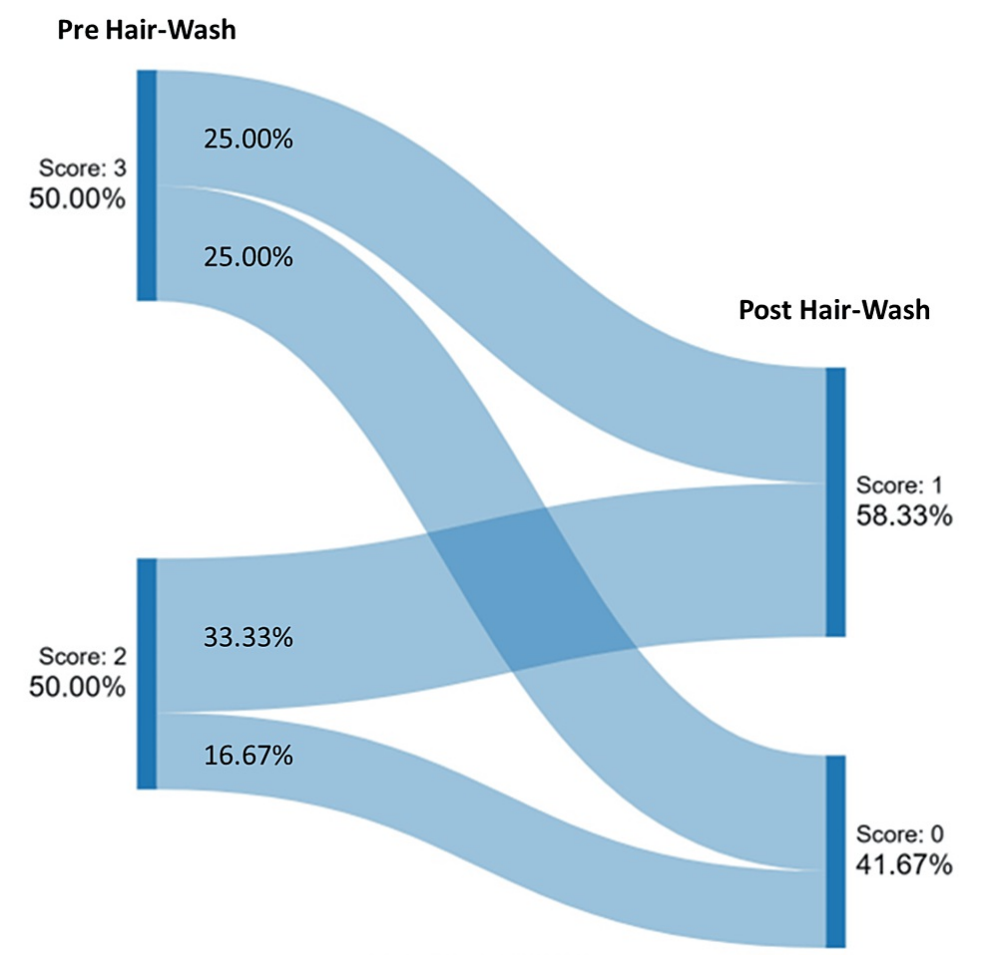
Change in non-adherent scalp flakes assessed by 60-second hair combing Scale used to assess the non-adherent/visible scalp flaking score: 0=almost none, 1=light flakes, 2=moderate flakes, 3=heavy flakes

**Figure 5:**
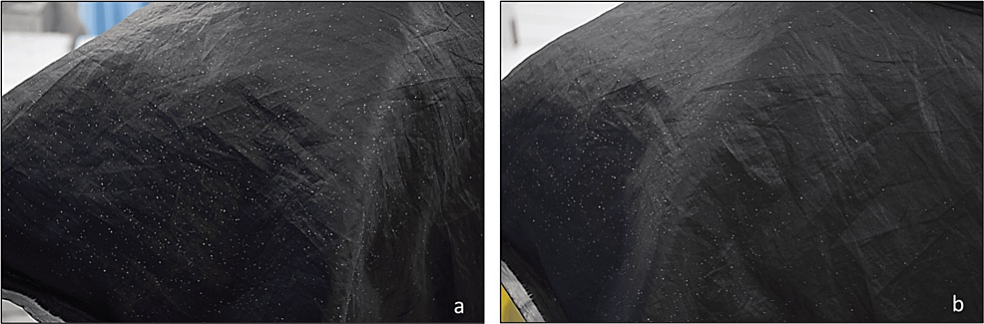
Shoulder area photograph for visible flakes assessed using a 60-second hair combing test; (a) pre-hair wash; (b) post-hair wash

Additionally, the 60-second hair combing assessment demonstrated a noteworthy reduction in non-adherent or visible flakes post-intervention. At baseline, the cohort was equally distributed between heavy and moderate visible flake scores. Post-hair wash, light flakes were observed in 58.33% of subjects, and no flakes were observed in 41.67% of subjects. Specifically, 41.67% of subjects saw a reduction by one level in the scoring of non-adherent flakes, while 68.33% saw a reduction by two levels. This indicates a reliable assessment of non-adherent flakes using the 60-second hair combing test (Figures 4, 5).

Association between ASFS and phototrichogram techniques

The chi-squared test revealed a highly significant association between the ASFS and phototrichogram analysis in the evaluation of scalp dandruff (p<0.001). This statistical analysis indicates a strong correlation between the two variables, suggesting that individuals with higher ASFS scores are more likely to exhibit certain scalp conditions in phototrichogram analysis and vice versa. Specifically, the scalp condition changed to reduce the amount of keratin, with dry scalp transitioning to normal scalp, as observed in the phototrichogram analysis. This association underscores the complementary nature of ASFS and phototrichogram techniques in assessing scalp dandruff and its associated scalp conditions (Table 2).

**Table 2:** ASFS and scalp condition association (chi-squared test)

Statistics	Value	df	Asymptotic significance (two-sided)
Pre-hair-wash results			
Pearson chi-squared	96	1	<0.001
N of valid cases	96		
Post-hair wash results			
Pearson chi-squared	192	4	<0.001
N of valid cases	96		

Furthermore, a similar trend was observed between non-adherent flakes measured by the 60-second hair combing test and adherent flakes measured by ASFS. The reduction in non-adherent flakes post-intervention corresponded with the decrease in adherent scalp flakes, indicating a consistent pattern of dandruff reduction across assessment methods.

Inter-evaluator and inter-operator variability assessments

The analysis of inter-evaluator variability for the ASFS demonstrated a substantial level of agreement among evaluators, with a Fleiss Multirater Kappa of 0.69 (p<0.0001). This high level of agreement underscores the reliability and consistency of ASFS assessments conducted by different evaluators, indicating minimal variability in the evaluation process. Similarly, the 60-second hair combing test exhibited substantial agreement among evaluators, as indicated by a Fleiss Multirater Kappa of 0.64 (p<0.0001). This finding suggests consistent and reliable assessment of non-adherent scalp flakes by different evaluators.

**Figure 6:**
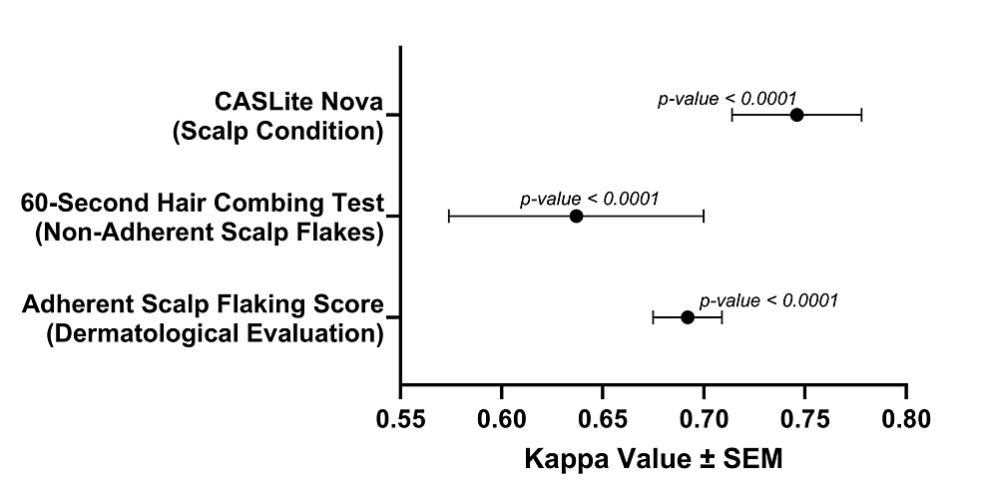
Fleiss Multirater Kappa values for different assessment methods

In the analysis of inter-operator variability for phototrichogram using CASLite Nova, an almost perfect agreement was observed among trained instrument operators, with a Fleiss Multirater Kappa of 0.75 (p<0.0001). This high level of agreement indicates minimal variability in scalp condition assessments conducted by different operators, highlighting the reliability and consistency of phototrichogram evaluations across multiple operators. These findings underscore the significance of comprehensive training in dermatological evaluation and instrumental operation, ensuring accurate and reproducible results with minimal variability (Figure 6).

## Discussion

The objectives of this study were twofold: first, to establish the correlation between three dandruff assessment methods, the ASFS, phototrichogram, and the 60-second hair combing test, before and after hair wash interventions in adult subjects. Second, to evaluate the inter-evaluator and inter-operator reliability of these assessment techniques. This study aimed to provide a comprehensive understanding of the effectiveness and reliability of these methods for assessing scalp dandruff.

The reliability of ASFS as an evaluation method has been well-documented in previous studies, making it a credible metric for assessing dandruff severity. The results of our study demonstrated significant changes from baseline in all three assessment parameters post-hair wash intervention. The ASFS showed a substantial reduction from 23.67±2.06 at baseline to 6.67±4.46 post-hair wash, indicating a mean reduction of 17.00±5.22 (71.40%, p<0.001). This significant decrease in ASFS scores suggests the effectiveness of hair wash in reducing adherent scalp flakes [12-14].

Similarly, phototrichogram has been established as a reliable instrument for detailed scalp assessments, providing objective and reproducible results. In our study, the phototrichogram analysis using CASLite Nova revealed significant improvements in scalp condition. More than 50% of the scalp zones with some keratin and nearly two-thirds of the scalp zones with much keratin transitioned to normal, good scalp condition post-hair wash. Overall, 60.42% of the total scalp zones assessed were found to be in normal and good condition post-hair wash compared to none at baseline [15-17].

The 60-second hair combing test was conducted to assess the non-adherent or visible flakes. At baseline, the cohort was equally distributed between heavy and moderate visible flake scores. Post-hair wash, light flakes were observed in 58.33% of subjects and no flakes in 41.67% of subjects. This indicates a significant reduction in non-adherent flakes, with 41.67% of subjects seeing a reduction by one level and 68.33% seeing a reduction by two levels. The 60-second hair combing test, a simple and effective method for assessing non-adherent flakes, thus validated that the findings are in line with ASFS and phototrichogram results of adherent scalp flakes. The similar trends observed across ASFS, phototrichogram, and the 60-second hair combing test post-hair wash intervention highlight the reliability of these evaluation methods. Each technique independently confirmed the reduction in dandruff severity, reinforcing the credibility of the assessment processes used in this study.

The chi-squared test results indicated a highly significant association between ASFS and phototrichogram analysis (p<0.001). This strong correlation suggests that individuals with higher ASFS scores are more likely to exhibit certain scalp conditions in phototrichogram analysis and vice versa. This finding aligns with existing literature that emphasizes the relationship between clinical assessment scores and instrumental evaluations in dermatological research [18]. The association observed in this study contributes to the validation of using either ASFS or phototrichogram for comprehensive dandruff assessment. Additionally, a similar trend was observed between non-adherent flakes measured by the 60-second hair combing test and adherent flakes measured by ASFS. The reduction in non-adherent flakes post-intervention corresponded with the decrease in adherent scalp flakes, indicating a consistent pattern of dandruff reduction across assessment methods.

In healthcare research, where the objective often revolves around improving treatment outcomes, the responsiveness and reliability of measurement tools are paramount. Understanding and ensuring inter-operator reliability is essential for maintaining consistency and accuracy in data collection across different individuals performing the same task. This reliability minimizes variability and bias that might arise from differences in interpretation by the evaluator or technique of the operator. The importance of comprehensive training from qualified dermatologists and professional instrument experts before conducting evaluations cannot be overstated. Such training ensures that evaluators and operators adhere to consistent standards, leading to reliable and accurate assessments [19].

The analysis of inter-evaluator variability for ASFS demonstrated a substantial level of agreement among evaluators, with a Fleiss Multirater Kappa of 0.692 (p<0.0001). This high level of agreement underscores the reliability and consistency of ASFS assessments conducted by different evaluators. Similarly, the 60-second hair combing test exhibited a substantial agreement among evaluators, with a Fleiss Multirater Kappa of 0.637 (p<0.0001). This finding suggests that the assessment of non-adherent scalp flakes by different evaluators was consistent and reliable. For phototrichogram evaluations using CASLite Nova, the inter-operator variability analysis also showed a substantial level of agreement among trained instrument operators, with a Fleiss Multirater Kappa of 0.746 (p<0.0001). This high level of agreement indicates minimal variability in scalp condition assessments conducted by different operators, emphasizing the reliability and consistency of these evaluations.

The significant correlations observed between the three methods, ASFS, phototrichogram, and the 60-second hair combing test, further validate their use in clinical and research settings. The high level of inter-evaluator and inter-operator agreement highlights the importance of comprehensive training in ensuring accurate and reproducible results. These findings contribute to the scientific understanding of scalp dandruff assessment and support the development of standardized methodologies for future research. However, this study has some limitations. The small sample size of 12 subjects may limit the generalizability of the findings. Additionally, the study was conducted in a single center, which may not account for variability across different populations and settings. Future studies with larger sample sizes, multi-center designs, and employing random sampling techniques are needed to better reflect the broader population and further explore the correlations between different dandruff assessment methods.

## Conclusions

This study underscores the effectiveness and reliability of three different methods, ASFS, phototrichogram using CASLite Nova, and the 60-second hair combing test, in assessing scalp dandruff. Significant reductions in dandruff were observed post-hair wash across all methods, establishing a clear trend and validating the reliability of these evaluation techniques. The strong association between ASFS and scalp condition assessed by phototrichogram, as well as between adherent and non-adherent flakes, further reinforces the interrelated nature of these assessment methods.

Furthermore, the study highlights the importance of comprehensive training for evaluators and instrument operators, demonstrating substantial inter-evaluator and inter-operator reliability. With Fleiss Multirater Kappa values indicating high agreement among trained personnel, the findings emphasize the necessity of standardized training to achieve consistent and accurate results. These conclusions not only validate the methods used but also provide a framework for future studies to ensure reliable and reproducible outcomes in scalp dandruff assessment.
